# Abdominal Pain in a Gender-Diverse Patient: A Standardized Patient Case for Second-Year Medical Students

**DOI:** 10.7759/cureus.77873

**Published:** 2025-01-23

**Authors:** Sarah Stumbar, Nahal Khamisani, Prasad Bhoite, Emiri Uchiyama, Maria Stevens, John M Saunders, Annette Davis, Nana Aisha Garba

**Affiliations:** 1 Medical Education, Florida International University Herbert Wertheim College of Medicine (FIU HWCOM), Miami, USA; 2 Medical School, Florida International University Herbert Wertheim College of Medicine (FIU HWCOM), Miami, USA; 3 Medical Education and Simulation, Florida International University Herbert Wertheim College of Medicine (FIU HWCOM), Miami, USA

**Keywords:** clinical skills education, lgbtq+, lgbtq medicine, patient-centered communication, sexual and gender minorities, transgender, transgender health and gender identity, undergraduate and graduate medical education

## Abstract

Introduction

With a growing emphasis on addressing the social determinants of health as a key component of patient-centered healthcare, holistically caring for marginalized groups such as the lesbian, gay, bisexual, transgender, and queer (LGBTQ+) population is an essential skill for future physicians. Yet, training on this topic remains limited in the undergraduate medical curriculum. Using gender-diverse individuals for standardized patient (SP) encounters presents a unique opportunity for students to interact with members of gender-diverse communities while practicing their inclusive patient interviewing skills.

Methods

This session consisted of a 1.5-hour small group during which students interviewed a sex and gender minority (SGM) SP presenting with abdominal pain. The patient encounter was followed by a debriefing session, during which the SP provided feedback and shared their lived experiences, including those involving the health care system. Students completed optional, anonymous, online, pre- and post-session surveys to assess their knowledge, perceived impact of, and satisfaction with the session.

Results

Around 95 of 104 (91%) students responded to both surveys. Results showed statistically significant increases in the number of students who stated that they were confident in their ability to take a sexual history, use terminology that is inclusive of different gender identities, and elicit health concerns frequently experienced by SGM people. Additionally, between the pre- and post-survey, there was a statistically significant increase in students’ identification of specific health disparities experienced by gender-diverse people.

Discussion

This case improved students’ knowledge regarding health disparities of gender-diverse people and their perception of their skills related to caring for this population. Direct assessment of students’ skills would allow for further assessment of the impact of this session.

Conclusion

This study demonstrates that a 1.5-hour faculty-facilitated small group SP encounter with a gender-diverse actor significantly improved second-year medical students' perceived confidence and competence in caring for SGM people. It also improved their understanding of health disparities. These outcomes are further reinforced by utilizing SGM individuals as SPs.

## Introduction

According to the Healthcare Equality Index, 70% of transgender people and 56% of lesbian, gay, or bisexual individuals reported at least one experience of discrimination when receiving healthcare [1]. A lack of high-quality undergraduate medical education regarding lesbian, gay, bisexual, transgender, and queer (LGBTQ+) health-specific topics may perpetuate health disparities [2]. In a survey at a single United Kingdom-based medical school, 85% of respondents reported a lack of LGBTQ+ health education in their curriculum, as well as low confidence in communicating with sexual and gender minority (SGM) patients [3]. Similarly, sexual history taking continues to be a topic of discomfort for medical providers when treating SGM patients [4]. In order to improve the quality of care that SGM patients receive, adequate training of medical students regarding the specific concerns of these diverse populations is essential.

However, there are various barriers to developing and implementing relevant curricula. In a 2018 survey of Family Medicine clerkship directors, only 24% reported that they were likely to invite an SGM patient to their course; and, similarly, only 27% reported access to a pre-existing curriculum [5]. In another Family Medicine clerkship director survey, a lack of expertise was cited as a barrier to content inclusion by 22% of respondents [6]. In an attempt to address these barriers, this study aimed to identify the outcomes of an easily implemented small group session regarding transgender healthcare for second-year medical students.

This article was previously presented as an oral presentation at the 2024 Society of General Internal Medicine (SGIM) Mid-Atlantic Regional Meeting on October 17, 2024.

## Materials and methods

Students at the Florida International University Herbert Wertheim College of Medicine (FIU HWCOM) have a longitudinal, integrated curriculum related to sexual and reproductive health, including sessions specific to LGBTQ+ health. The learning objectives of this curriculum have been previously published [7]. A two-hour session in the first year introduces students to the concepts of sex, gender, and sexual orientation, among others, and discusses health disparities experienced by the LGBTQ+ community. In the second year, an additional two-hour session discusses the social determinants of sexual and reproductive health and includes a patient panel of people with diverse sexual orientations and gender identities; the structure, materials, and outcomes of this session have been previously published on the MedEdPORTAL® (www.mededportal.org) [8]. During the second-year clinical skills course, students receive an additional one-hour lecture on strategies for taking an inclusive and holistic sexual history; the structure and outcomes of this session, as delivered in a virtual format, have been previously published [9]. Following the lecture on sexual history, students participate in a 1.5-hour faculty-facilitated small group interview of an SGM standardized patient (SP). Prior to the small group session with the students, faculty participate in a 30-minute training with an expert with experience caring for SGM patients. During the faculty development session, the faculty guide is reviewed in detail, and the faculty are given the opportunity to ask any questions that they have about leading the session.

Taking turns to complete the interview, students initially take a full health history of the SP with a focus on the sexual history. Each student is allowed to “tag out” to a classmate as needed or desired; the faculty member can also opt to stop the student and call on another student to continue the interview. Students may be stopped once they complete obtaining the history or if they request further guidance on how to proceed from the faculty or their peers. After the interview is completed or 25 minutes have passed (whichever is sooner), the session then moves into an in-depth 30-minute debrief of the encounter. This part of the session is facilitated in such a way as to allow the SP to discuss his/her/their own experiences in healthcare as an SGM individual and to provide feedback to the students regarding their history-taking skills. Additionally, students are encouraged to reflect on what went well, what could be improved, and how they felt during the patient interview. As time permits, the debrief is moved into discussing unique health concerns of the SGM community, such as experiences with sexual violence, intimate partner violence, self-treatment with hormones, gender affirmation surgery and use of silicone, mental health, social support, HIV prevention, organ-specific cancer screenings, and updating legal documents. The debrief concludes with a discussion on how to improve patient-centered communication to create a more welcoming environment for SGM patients.

For the purpose of this study, students interviewed an SP presenting with abdominal pain. The patient encounter was followed by a debriefing session during which the SP provided feedback and shared their lived experiences, including those involving the healthcare system. 

To evaluate the session, students were given online, optional pre- and post-session surveys consisting of five-point Likert-type responses, several structured questions, and one unstructured short-answer question. The pre- and post-session surveys both included the same three Likert-type questions and two structured questions. These questions assessed students’ perceptions of their ability to take a sexual history, use terminology that is inclusive of different gender identities, and elicit health concerns frequently experienced by transgender people. The post-session survey also included six additional Likert-type questions and one unstructured question. The post-survey Likert-type questions assessed the degree to which students felt the workshop helped increase their knowledge and skills regarding the effective provision of medical care for SGM patients and helped them identify their own biases when providing care to this population. The two structured questions assessed the following: the students’ actual knowledge of the five components of the "5Ps" model of sexual history, including partners, practices, protection from sexually transmitted infections (STIs), past history of STIs, and pregnancy intention, as well as of the health concerns frequently experienced by SGM people [10]. The only unstructured question asked students to identify the portion of the session they found most valuable.

For data analysis, R programming language version 3.6.3 (R Foundation for Statistical Computing, Vienna, Austria) and Microsoft Excel (Microsoft Corp., Redmond, US) were used to analyze the quantitative and qualitative data, respectively. The pre- and post-session quantitative survey data was imported to R, and then exploratory data analysis was performed to determine the sample size, missing values, duplicate responses, and variable selection. A Wilcoxon signed-rank test was used to determine statistical significance between students’ five Likert-type responses to the three paired pre- and post-session survey questions. Frequency distribution was performed for the responses to the six post-session only questions and the unstructured questions. For the two structured questions, students’ responses were scored and mean scores were calculated for the pre- and post-session data, after which a paired t-test was used to determine statistical significance. For the unstructured question requiring free text responses, standardized codes were created by the subject matter experts (two clinical faculty), and each student’s response was coded respectively with content analysis. The Florida International University Institutional Review Board provided exempt status to this project.

## Results

Around 104 members of the class of 2024 participated in the session, out of which 95 (91%) responded to both the pre- and post-session surveys. Analysis of students’ responses showed statistically significant increases in the post-session number of students who stated that they were confident in their ability to take a sexual history, use terminology that is inclusive of different gender identities, and elicit health concerns frequently experienced by transgender people compared to the pre-session (Table 1).

**Table 1:** Distributions of students’ ranked responses to the pre- and post-survey Ranked students’ responses to pre-and post-session questions (N=95). The data has been represented as mean±SD; the p-value is considered significant at p<0.05. Rank is determined from an evaluation of the pre- and post-survey responses on a five-point Likert scale, where 1=strongly disagree, 2=disagree, 3=neutral, 4=agree, and 5=strongly agree.

Question	Pre-Survey Mean Rank (±SD)	Post-Survey Mean Rank (±SD)	P-value (95% CI)
I am confident in my ability to take a sexual history.	3.7 (0.7)	4.3 (0.7)	<0.001 (-1.00,-0.99)
I am able to use terminology that is inclusive of different gender identities.	3.6 (0.8)	4.3 (0.6)	<0.001 (-1.49,-1.00)
I am able to take a patient history that elicits health concerns frequently experienced by transgender people.	3.1 (0.8)	4.2 (0.6)	<0.001 (-1.50,-1.49)

As shown in Table 2, 95% of respondents agreed or strongly agreed that the session increased their skills to effectively provide medical care for transgender patients, while 5% were neutral. About 96% of respondents agreed or strongly agreed that the workshop experience increased their knowledge about how to effectively provide medical care for transgender patients, while only 4% remained neutral. About 79% of respondents either agreed or strongly agreed that they were confident in their ability to screen patients for intimate partner violence, while 18% were neutral and 3% disagreed. About 94% of respondents agreed or strongly agreed that the standardized patient case was realistic and believable, while 6% were neutral.

**Table 2:** Percentage distribution of students’ responses to the post-session survey The data has been represented as N (%); the p-value is considered significant at p<0.05.

Question	Strongly Agree or Agree (%)	Neutral (%)	Strongly Disagree or Disagree (%)
The experience today increased my skills to effectively provide medical care for transgender patients.	90 (95)	5 (5)	0
The experience today increased my knowledge about how to effectively provide medical care for transgender patients.	91 (96)	4 (4)	0
The standardized patient case was realistic and believable.	89 (94)	6 (6)	0
The standardized patient provided valuable feedback during our post-encounter debriefing session.	89 (94)	6 (6)	0
The standardized patient case post-encounter debriefing helped me to identify my own biases in providing care to transgender patients.	89 (94)	6 (6)	0
I am confident in my ability to screen patients for intimate partner violence.	75 (79)	17 (18)	3 (3)

Assessment of students’ knowledge of health concerns frequently experienced by SGM people found a statistically significant increase in the mean score obtained post-session compared to the pre-session survey, with a p-value at 95% confidence interval <0.001. However, the increase in students’ knowledge of the five components of sexual history from before to after the session was not statistically significant at a 95% confidence interval (p=0.63). The results of the qualitative analysis from the one unstructured question highlighted four major themes. Responses showed that students valued the experience of gathering history from an SGM community member the most, followed by the post-session debrief and feedback (Table 3).

**Table 3:** Aspects of the session that students found most valuable, with selected supportive quotes (N=95) SP: standardized patient; SGM: sex and gender minority

Themes	Number of Students Who Selected Each Theme As Most Valuable	Select Quotes
Observing/practicing or gathering history from an SGM patient with lived experiences	72	“Being able to interact and take a history from a transgender patient was invaluable. I got to practice my skills in a situation that I have not really encountered and I learned so much from it. It is one thing to know the questions to ask, but actually being able to ask them to a patient is so important and more difficult than I thought it would be. I think more experiences like this would be super helpful.”
Post-session debrief/feedback	46	“I found the interview and dialogue with the SP following the visit to be most insightful. It also helped me to reevaluate and become more conscious of my own biases.”
Having a fourth-year medical student facilitate the session	3	“I feel like we had a very valuable and open conversation with the SP and the fourth-year medical student.”
Interaction with the facilitator	1	“The interaction with the patient and the facilitator.”

## Discussion

While a dearth of high-quality LBGTQ+ training materials at the undergraduate medical education level may perpetuate health disparities experienced by this community, sessions such as the one presented here aimed to fill this curricular gap [2]. The results of our study suggest that students felt like the session effectively increased their confidence regarding their ability to take a sexual history, use inclusive language, and elicit health concerns that are particularly relevant to this patient population. This was supported by a statistically significant increase in the students’ ability to list five health concerns of transgender patients when comparing pre- and post-session responses. With adequate training in interacting with SGM community members and high satisfaction scores from students, clerkship directors are more likely to invite an SGM patient to this curriculum. Clerkship directors may feel more comfortable inviting an SGM patient to teach if they have this curriculum to support the implementation of the session. However, there was no statistically significant increase in students’ ability to list the 5Ps sexual history model between the pre- and post-session surveys; this may be due to high levels of knowledge on this topic prior to the session.

On a Likert-type evaluation, students reported that the session increased their knowledge and skills related to caring for SGM patients and that it helped them to identify their own biases. Student feedback about the session overall was very positive. They overwhelmingly reported that the SP case was believable and that the SPs provided valuable feedback. Students also identified the parts of the session that enabled them to directly interact with the SP - either through the patient interview scenario or receipt of feedback during the debriefing - as being the most valuable. This suggests that having people who identify as SGM serve as SPs enhanced the session by cultivating a believable narrative and providing students the opportunity to interact with community members with real, lived experiences. The debriefing session after the SP interview allowed not only for feedback about student performance but also for the sharing of stories about positive and negative healthcare experiences by the SPs. This sharing added believability, context, and relevance to the LGBTQ+ health disparities and concerns presented in this session. Furthermore, the shared stories and experiences humanize the patients and consolidate student learning.

A major limitation of the evaluation approach presented here is that students notoriously over-report their skill and confidence levels, which therefore does not equate to competency. Additionally, the study design does not allow for causal conclusions to be drawn as there is no control group. The study also focuses on immediate outcomes and long-term impacts are unknown. Additionally, these results may be challenging to generalize to other schools as the structure and content of LGBTQ+ and sexual health curricula vary greatly across curricula, with the session coming toward the end of our institution’s pre-clinical coverage of related topics.

This session has evolved significantly over the past six years. It began as a one-hour lecture-only format; for the subsequent two years, cisgender actors played the transgender SP role. This, of course, perpetuated concerns for bias and stereotyping and did not allow for students to experience the real, lived experiences of transgender patient actors. However, we experienced significant challenges in recruiting SGM actors; many were unable to take time off work to participate in the session. While we were offering a small stipend to participate and make up for lost wages, many potential participants were wary of the extensive paperwork required by our state institution to receive payment. Additionally, many potential participants identified concerns about the intentions of the session, potential bias and discrimination, and the need to come to campus. In 2020, the COVID-19 pandemic necessitated a virtual format for this session, and we were easily able to recruit transgender, non-binary, and gender-diverse participants. Many voiced that they were comfortable with the knowledge that they could just disconnect from the session if they were ever uncomfortable or discriminated against, and fortunately, this was never necessary. After a successful virtual run in 2020, we have been able to recruit SGM people for subsequent sessions. The evolution of this session has taught us the importance of coalition building for the success of similar sessions teaching about marginalized populations, as well as the need to address social determinants, such as lost wages, fear of discrimination, and transportation barriers.

There are no current plans to make substantial changes to this session. However, in 2024, an observed structured clinical examination (OSCE) was piloted to enable us to better assess the effectiveness of the curriculum. The rubric for the OSCE includes an assessment of student communication skills as they relate to SGM patients, preventive health recommendations, and history-taking skills. Future studies analyzing OSCE performance will be used to improve future iterations of the workshop presented here. 

## Conclusions

Student feedback about this session, which combines a lecture with a small-group session where students interview SGM patients, was positive. Students overwhelmingly reported that the case was believable and that the SP provided valuable feedback. This suggests that having SGM people serve as SPs enhanced the session by allowing students to interact with community members with lived experiences. The sharing of stories added believability, context, and relevance to LGBTQ+ health disparities. The ability to practice interviewing SGM patients was most frequently cited as the aspect of the session that the students found to be the most valuable, followed by the post-session debrief and feedback. 

Our experiences with this session have taught us the need to address social determinants, such as lost wages, fear of discrimination, and transportation barriers, when including community members in our teaching about marginalized populations. While students perceived that the session improved a variety of their specific communication skills, a limitation of the self-evaluation approach presented here is that students over-report their level of skills and confidence. Therefore, we have recently incorporated an OSCE into the curriculum that requires students to assess an SGM patient presenting to establish care. This OSCE allows for direct assessment of students’ skills and presents future directions for this work.
